# Long-read and short-read RNA-seq reveal the transcriptional regulation characteristics of *PICK1* in Baoshan pig testis

**DOI:** 10.1590/1984-3143-AR2024-0047

**Published:** 2024-10-04

**Authors:** Xia Zhang, Hailong Huo, Guowen Fu, Changyao Li, Wan Lin, Hongmei Dai, Xuemin Xi, Lan Zhai, Qingting Yuan, Guiying Zhao, Jinlong Huo

**Affiliations:** 1 Department of Biological and Food Engineering, Lyuliang University, Lvliang, Shanxi, China; 2 College of Animal Science and Technology, Yunnan Agricultural University, Kunming, Yunnan, China; 3 Yunnan Open University, Kunming, Yunnan, China; 4 Yunnan Vocational and Technical College of Agriculture, Kunming, Yunnan, China; 5 College of Veterinary Medicine, Yunnan Agricultural University, Kunming, Yunnan, China

**Keywords:** Baoshan pig (BS), PICK1, long-read and short-read RNA-seq, transcriptional regulatory

## Abstract

*PICK1* plays a crucial role in mammalian spermatogenesis. Here, we integrated single-molecule long-read and short-read sequencing to comprehensively examine *PICK1* expression patterns in adult Baoshan pig (BS) testes. We identified the most important transcript ENSSSCT00000000120 of *PICK1*, obtaining its full-length coding sequence (CDS) spanning 1254 bp. Gene structure analysis located *PICK1* on pig chromosome 5 with 14 exons. Protein structure analysis reflected that PICK1 consisted of 417 amino acids containing two conserved domains, PDZ and BAR_PICK1. Phylogenetic analysis underscored the evolutionary conservation and homology of PICK1 across different mammalian species. Evaluation of protein interaction network, KEGG, and GO pathways implied that interacted with 50 proteins, predominantly involved in glutamatergic synapses, amphetamine addiction, neuroactive ligand-receptor interactions, dopaminergic synapses, and synaptic vesicle recycling, and PICK1 exhibited significant correlation with DLG4 and TBC1D20. Functional annotation identified that PICK1 was involved in 9 GOs, including seven cellular components and two molecular functions. ceRNA network analysis suggested BS *PICK1* was regulated by seven miRNA targets. Moreover, qPCR expression analysis across 15 tissues highlighted that *PICK1* was highly expressed in the bulbourethral gland and testis. Subcellular localization analysis in ST (Swine Tesits) cells demonstrated that PICK1 significantly localized within the cytoplasm. Overall, our findings shed new light on *PICK1*’s role in BS reproduction, providing a foundation for further functional studies of *PICK1*.

## Introduction

The Baoshan pig (BS), originating from the Baoshan region of Yunnan Province, China, is esteemed among indigenous pig breeds in southern China owing to its extensive historical lineage. Renowned for its moderate body size, gentle temperament, strong adaptability, moderate growth rate, even muscle and fat distribution, and excellent meat quality. BS has effectively contributed to rural economic development and enhanced the vitality of animal husbandry (Diao et al., 2019; Li et al., 2024). Studying the reproductive traits of the BS breed will provide essential data for formulating breeding strategies and guiding its development and utilization, ultimately contributing to local economic growth.

PICK1, also known as PRKCA1 (Protein Kinase C Alpha 1), is a peripheral membrane protein initially identified through the yeast two-hybrid system. It exhibits specific interaction with the catalytic domain of PKC and serves as a potent substrate for PKC phosphorylation both in vitro and in vivo (Staudinger et al., 1995). Furthermore, *PICK1* plays a pivotal role in sperm development, contributing to various aspects of spermatogenesis, including sperm maturation, motility, and fertilization. The acrosome, a sac-like organelle containing hydrolytic enzymes, covers the front of the sperm nucleus and is situated between the nucleus and the plasma membrane (Ikawa et al., 2010). During fertilization, *PICK1* is pivotal when the sperm interacts with the zona pellucida. A homozygous missense mutation (G198A) in exon 13 of the *PICK1* gene was identified in a Chinese family, resulting in an acrosome defect in sperm (Liu et al., 2010). Studies revealed the importance of *PICK1* in acrosomal granule transport from the Golgi apparatus to the acrosome. Male mice lacking *PICK1* exhibit a globozoospermia phenotype resembling humans, characterized by early acrosome fragmentation during spermatogenesis, leading to male infertility due to reduced sperm count, and impaired sperm vitality (Xiao et al., 2009). In addition, *PICK1* is also involved in the progression of various inflammation-related diseases. Its deficiency impairs autophagic function, exacerbating sepsis-induced acute lung injury (Mo et al., 2018). In sepsis-associated encephalopathy, PICK1 forms a complex with TLR4, exerting a protective effect against inflammatory damage (Wang et al., 2021). Additionally, PICK1 mitigates lipopolysaccharide-induced apoptosis in renal tubular epithelial cells, protecting against sepsis-related acute kidney injury (Dou et al., 2021).

Integrating high-throughput long reads from third-generation sequencing (PacBio) with short reads from second-generation sequencing (Illumina) enables a more comprehensive capture of transcript full-length information, facilitating the identification of new transcripts, splice variants, and non-coding RNAs, thereby enhancing our insight on gene expression mechanisms (Hu et al., 2021). Here, we employed Pacific Biotechnology isoform sequencing (PacBio Iso-Seq) and Illumina RNA sequencing (RNA-seq) technologies to assess the expression of *PICK1* mRNA and alternative splicing, as well as related to miRNA, and lncRNA in BS testis. We analyzed the molecular characteristics of the *PICK1*, and its corresponding protein functions, and conducted protein-protein interaction. Additionally, we constructed a competitive endogenous RNA (ceRNA) regulatory network by annotating the *PICK1* gene and identified associated GO terms, miRNAs, and IncRNAs. This study underlines the significance of *PICK1* in the testis, providing a valuable resource for further study of the mechanisms and functions of the *PICK1* gene in BS spermatogenesis.

## Methods

### Long-read and short-read RNA-seq Iso-seq sequencing

Three 12-month-old adult BS boars were chosen, and their testis samples were obtained by surgical castration. All animal procedures were approved by the Research Ethics Committee of Yunnan Agricultural University (No. YNAUREC2023628). We conducted transcriptomic analysis of *PICK1* in BS testes using an integrated approach, combining long-read Iso-seq sequencing with short-read RNA-seq sequencing. Long-read sequencing data were obtained from Wuhan GrandOmics Co., Ltd. China, while short-read sequencing data were obtained from Tianjin Novogene Co., Ltd., China. Unannotated transcripts were merged with annotated transcripts from the Ensembl database to generate a comprehensive annotation file. Filtering and processing of second-generation sequencing data, coupled with the incorporation of newly identified subtypes from third-generation sequencing, facilitated the generation of BAM files. Visualization of *PICK1* gene transcripts was achieved using the Sashimi Plot function in IGV. Subsequently, the pig reference genome (Sus scrofa 11.1) index was constructed using STAR-2.5.2. Gene expression quantification, comprising raw expression levels and normalized expression values (TPM), was calculated using FeatureCounts-2.0.1 and Salmon-1.5.1, respectively. Finally, the R package Gviz was employed to visualize the expression abundance of the *PICK1* gene.

### Transcript amplification and sequence determination

Primers F1/R1 (F1: ACTCTCGGAACCATGTTTGCAG; R1: CTCAGGAGTCACACCAGCTTC) were designed to amplify the transcript ENSSSCT00000000120 using Premix Taq™ (Takara, Dalian, China). The total reaction volume of 25 μL comprised 12.5 μL of Premix, 1 μL each of 10 μM F1/R1 primers, 1 μL of 50 ng/μL testis cDNA, and the remaining volume was filled with H_2_O. The amplification program consisted of initial denaturation at 95°C for 5 min; followed by 35 cycles of denaturation at 95°C for 30 sec, annealing at 61°C for 30 sec, extension at 72°C for 80 sec; and a final extention at 72°C for 10 min.

### Characteristics analysis of transcript ENSSSCT00000000120

To obtain the complete coding sequence (CDS) of *PICK1* transcript ENSSSCT00000000120, we utilized Lasergene 7.1 to analyze the sequencing data. To decipher the feature information of PICK1 we used ProtParam to estimate the molecular weight, molecular formula, and isoelectric point. We parsed the functional domain, secondary structure, tertiary structure, hydrophobic structure, transmembrane helices, and signal peptide of the PICK1 protein using the SMART, SOPMA, I-TASSER, ProtScale, TMHMM, and SignalP websites, respectively. Finally, we measured the evolutionary relationship of PICK1 amino acid sequences across different species using MEGA11.

### Protein-protein interaction analysis of PICK1

We constructed the protein-protein interaction network using String 11.5. Additionally, we employed GO and KEGG functional enrichment analysis for these proteins using the R package clusterProfiler. In our analysis, entries with a significance threshold of *P*< 0.05 were considered statistically significant. Finally, we associated the identified proteins with gene expression obtained from transcriptome sequencing data and calculated the expression correlation between them.

### Regulatory network analysis of *PICK1*

We utilized the annotation of the UniProt database to acquire insights into the biological processes associated with *PICK1*, including cellular components and molecular function. To capture miRNAs and lncRNAs regulating *PICK1*, we analyzed the transcriptome data using miRanda 3.3 and RNAhybrid 2.1.2. Further, we visualized the ceRNA (competing endogenous RNA) transcriptional regulatory network using Cytoscape 3.9.1.

### Multi-tissue expression analysis of the *PICK1* gene

Fluorescent quantitative primers F2R2 (F2: TCCCTGGACATCGTGTTGAAG; R2: CTTGACAAGCCCATCATTGCAC) were designed using *PICK1* mRNA as a templet, with the housekeeping gene GAPDH serving as an internal reference (F3: CCTTCATTGACCTCCACTACATGGT; R3: CCACAACATACGTAGCACCAGCATC). The mRNA expression of *PICK1* was evaluated across 15 different tissues in BS. Data analysis followed the relative quantification 2-^ΔΔct^ method (Rao et al., 2013).

### Subcellular localization detection of PICK1

We constructed the pEGFP-C1-*PICK1* eukaryotic expression recombinant plasmids and transfected them into ST cells to locate the PICK1 and non-transfected ST cells, and those cells transfected with the EGFP-C1 vector served as negative controls. Subsequently, the nuclear and mitochondria of ST cells were stained using blue Hoechst 33342 and red MitoTracker, respectively. Finally, the expression and localization of PICK1 in ST cells were captured using inverted fluorescent microscopy.

## Results

### Alternative splicing of *PICK1*

**Figure 1:**
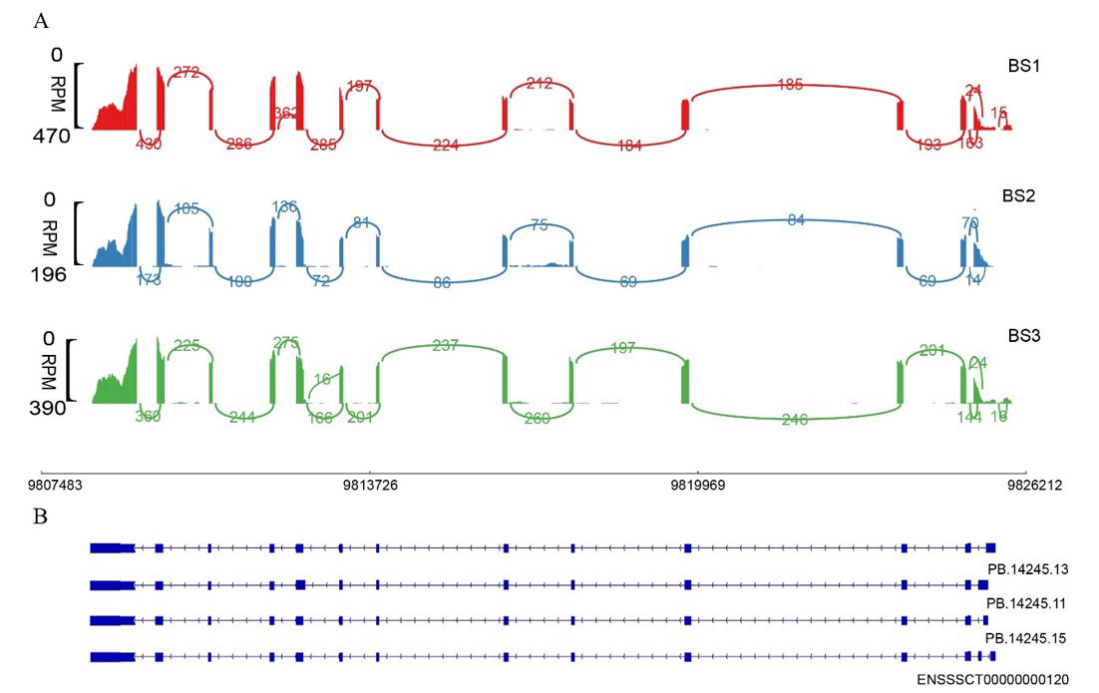
Transcripts and expression levels of *PICK1* from three testes of BS pigs.

Long-read and short-read sequencing results revealed that the distinct alternative splicing isoforms in three testes of BS pigs (Figure 1A), Specifically, novel transcripts PB.14245.13, PB.14245.11, and PB.14245.15 transcripts were identified from third-generation sequencing data. Notably, ENSSSCT00000000120 emerged as the predominant transcript, as shown in Figure 1B.

### *PICK1* gene expression characteristics

**Figure 2:**
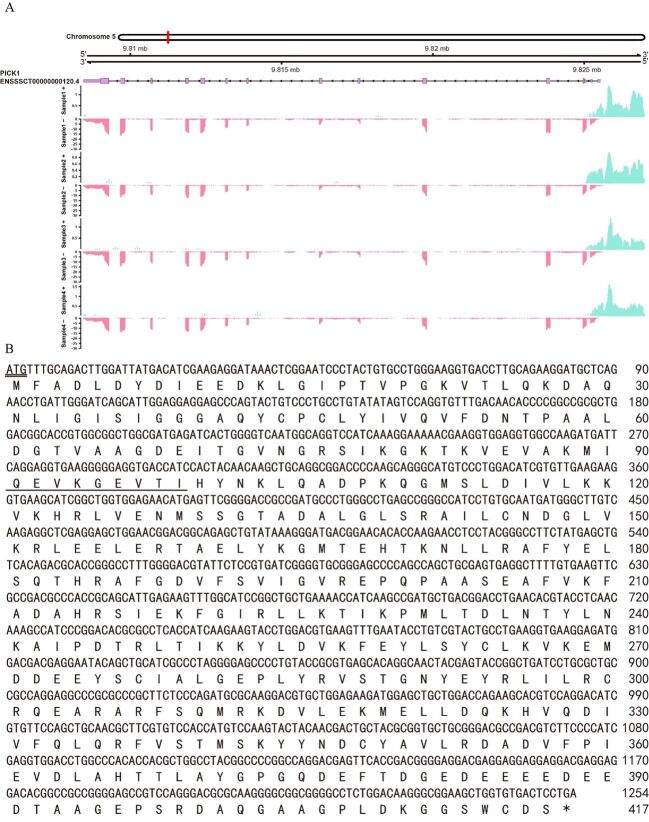
Gene structure of *PICK1*. (A) Chromosome location, exon, and intron abundance based on transcriptome sequencing; (B) *PICK1* gene coding sequence and amino acid sequence.

The average expression value of the transcript ENSSSCT00000000120 of the *PICK1* gene in BS pig testes was 1632.25, and was located on chromosome 5, with a total length of 17,089 bp. Gene annotation conducted by Gviz delineated transcript ENSSSCT00000000120 comprises 14 exons and 13 introns, with consistently high expression across all three BS pig samples (Figure 2A). A 1267 bp fragment of the *PICK1* gene was obtained Using primers F1/R1. Subsequent Sanger sequencing unveiled the full-length coding sequence (CDS) of *PICK1* as 1254 bp, encoding a total of 417 amino acids (Figure 2B).

### Protein sequence and structure of PICK1

**Figure 3:**
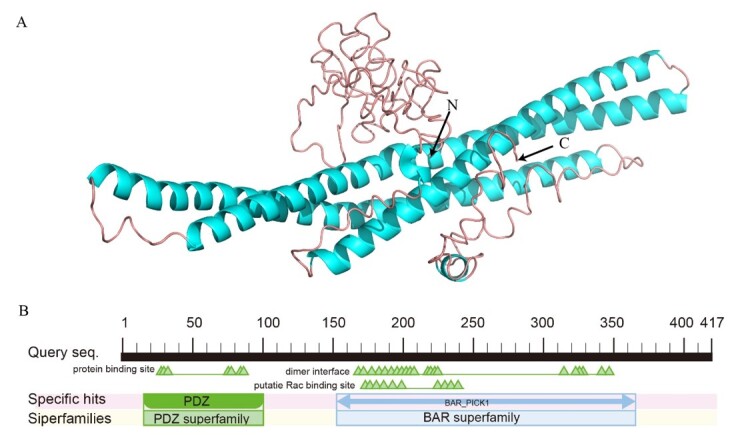
PICK1 protein spatial structure. (A) Tertiary structure of PICK1 protein; (B) Conserved domain of PICK1 protein.

The BS pig PICK1 protein had a molecular weight of 46.72 kD, characterized by a molecular formula of C_2063_H_3274_N_554_O_644_S_18_ and an isoelectric point of 5.02. It comprised 69 residues with negative charges and 52 residues with positive charges. Notably, amino acids at positions 49 and 190 exhibited maximum hydrophobicity values of 1.733, while those at positions 386 and 387 displayed minimum hydrophobicity values of -3.500. The N-terminus exhibited hydrophobicity, whereas the C-terminus demonstrated hydrophilicity. Although PICK1 contained the phosphorylation site of the enzyme, it lacked both a signal peptide and transmembrane structure. Regarding its secondary structure, The PICK1 protein from BS pigs predominantly comprised α-helix (56.59%, 236 amino acids), followed by the random coil (26.14%, 109 amino acids), the extended strand (11.75%, 49 amino acids), and β-turn (5.52%, 23 amino acids). The tertiary structure of the PICK1 protein was closely similar to the secondary structure composition, comprising four primary structural types: α-helix, random coil, extended chain, and β-turn (Figure 3A). Additionally, the PICK1 protein contained two conserved structural domains: PDZ and BAR_PICK1 (Figure 3B).

### Homology analysis of PICK1 proteins across species

**Figure 4:**
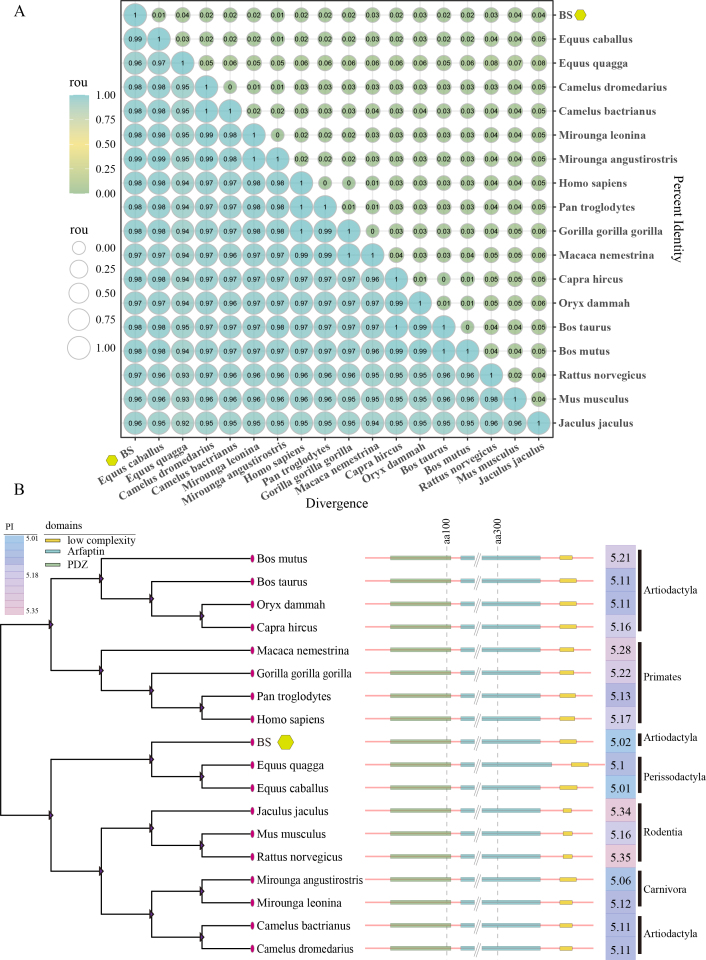
PICK1 proteins homology across different species. (A) Similarity; (B) Phylogenetic tree.

Multiple sequence alignment of PICK1 from various mammalian species revealed a remarkable amino acid sequences similarity exceeding 95% among BS pig, horse *(Equus caballus),* zebra *(Equus quagga)*, dromedary camel *(Camelus dromedarius)*, Bactrian camel *(Camelus bactrianus)*, southern elephant seal *(Mirounga leonina)*, northern elephant seal *(Mirounga angustirostris*), human *(Homo sapiens)*, chimpanzee *(Pan troglodytes)*, western lowland gorilla *(Gorilla gorilla gorilla)*, pig-tailed macaque *(Macaca nemestrina)*, goat *(Capra hircus)*, scimitar-horned oryx *(Oryx dammah)*, domestic cow *(Bos taurus)*, wild yak *(Bos mutus)*, rat *(Rattus norvegicus)*, mouse *(Mus musculus)*, and African jerboa *(Jaculus jaculus)* (Figure 4A). Phylogenetic analysis suggested clustering patterns: pigs with horses and zebras; domestic cattle, wild yaks, scimitar-horned oryx, and goats together; pig-tailed macaques, gorillas, chimpanzees, and humans together; lesser Egyptian jerboas, rats, and mice together; and northern elephant seals, southern elephant seals, Bactrian camels, and dromedary camels together. Further, phylogenetic analysis indicated that the PICK1 protein harbored identical functional domains across 18 species (Figure 4B).

### Protein-protein interaction

**Figure 5:**
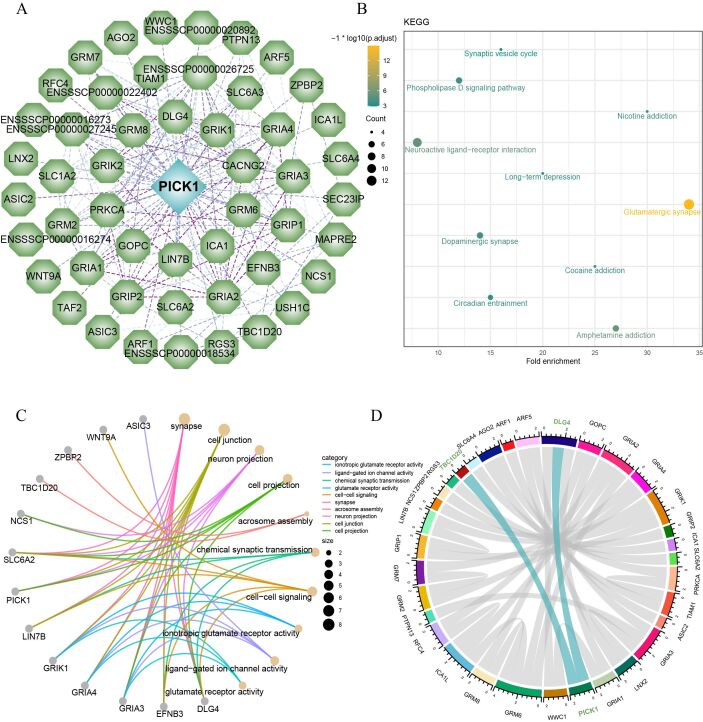
Protein interactive analysis. (A) PICK1 protein interaction network; (B) KEGG enrichment analysis of interacting proteins; (C) GO enrichment analysis of interacting proteins; (D) *PICK1* correlation chord plot.

The protein-protein interaction analysis revealed 50 proteins potentially interacted with PICK1 (Figure 5A). Subsequent KEGG enrichment analysis indicated that these proteins were mainly involved in pathways such as glutamatergic synapse, amphetamine addiction, neuroactive ligand-receptor interaction, dopaminergic synapse, phospholipase D signaling pathway, nicotine addiction, circadian entrainment, cocaine addiction, long-term depression, and synaptic vesicle cycle (Figure 5B). Further, GO enrichment analysis indicated that these proteins were primarily involved in synaptic processes, cell junctions, neuron projection, cell projection, acrosome assembly, chemical synaptic transmission, cell-cell signaling, ionotropic glutamate receptor activity, ligand-gated ion channel activity, and glutamate receptor activity (Figure 5C). Finally, we matched these proteins with gene expression data from BS pigs and identified significant correlations between PICK1 and DLG4, as well as TBC1D20, with correlation coefficients of 0.99954 and 0.99875, respectively (Figure 5D).

### ceRNA regulatory network of *PICK1*

**Figure 6:**
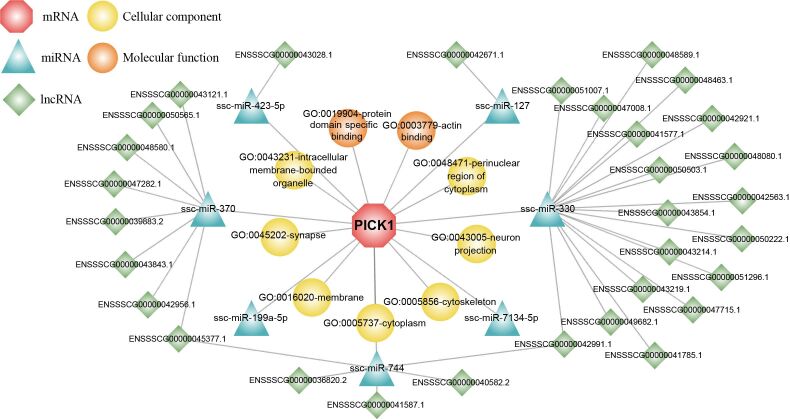
The functional annotation *PICK1* potential ceRNA regulatory network.

Functional annotation of *PICK1* revealed its involvement in various cellular components, including neuron projection, perinuclear region of cytoplasm, synapse, cytoskeleton, membrane, cytoplasm, and intracellular membrane-bounded organelle; in terms of molecular function, *PICK1* primarily involved in protein domain specific binding and actin binding. Additionally, seven miRNAs have been identified as primary regulators of porcine PICK1, namely ssc-miR-127, ssc-miR-330, ssc-miR-7134-5p, ssc-miR-744, ssc-miR-199a-5p, ssc-miR-370, and ssc-miR-423-5p. Among them, one, eighteen, five, eight, and one lncRNA competed with ssc-miR-127, ssc-miR-330, ssc-miR-744, ssc-miR-370, and ssc-miR-423-5p for binding to *PICK1*, respectively (Figure 6).

### Expression pattern of *PICK1* across multi-tissue

**Figure 7:**
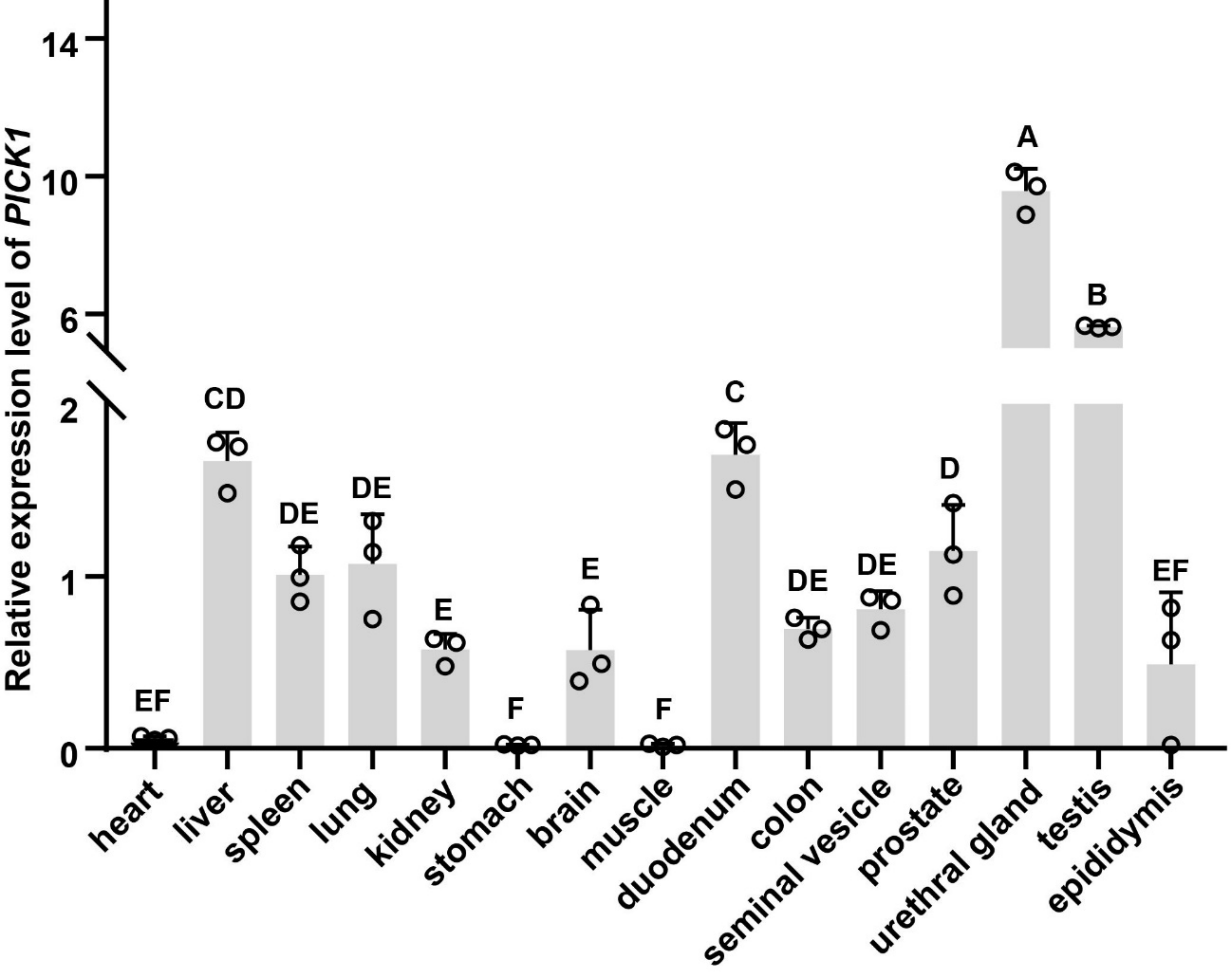
The multi-tissue expression pattern of the *PICK1* gene. Different capital letters represent significant differences (*P*<0.01).

Multi-tissue qPCR analysis revealed that the relative expression of *PICK1* displayed the highest level in the urethral glands of BS pigs, followed by the testis. Conversely, relatively low expression levels were observed in the colon, duodenum, liver, prostate, lung, spleen, seminal vesicle, kidney, and brain. Expression levels were almost negligible in the epididymis, heart, stomach, and muscle (Figure 7).

### Subcellular localization results of PICK1

**Figure 8:**
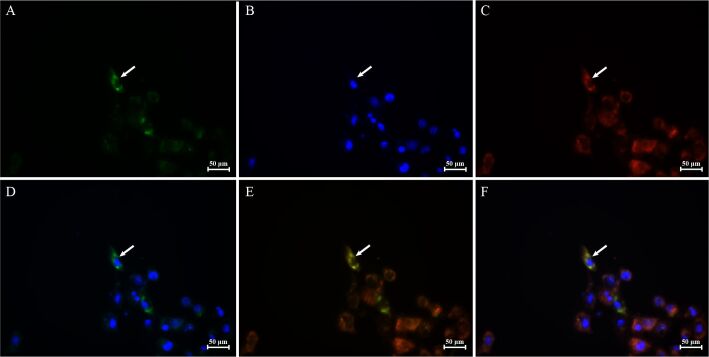
The subcellular localization results of PICK1 protein. (A) PICK1 protein; (B). nucleus; (C) mitochondria; (D) GFP merged with nucleus; (E) GFP merged with mitochondria; (F) superposition of GFP, nucleus, and mitochondria. Green, blue, and red represent PICK1, nucleus, and mitochondria, respectively.

Subcellular localization analysis indicated that the majority of the PICK1 protein resided in the cytoplasm of ST cells, with a minor fraction detected in the nucleus (Figure 8), consistent with the PSORT website prediction results, which estimated 89% of PICK1 in the cytoplasm.

## Discussion

Here, we conducted an in-depth analysis of the *PICK1* transcriptome in BS pig testis using a comprehensive approach involving long-read Iso-seq sequencing and short-read RNA-seq sequencing. Our analysis unveiled four prominently expressed isoforms in BS pig testis. Notably, ENSSSCT00000000120 emerged as the most significant isoform, boasting the highest number of exons. Consequently, we proceeded with our further analysis of this specific transcript. The complete coding sequence (CDS) of the *PICK1* gene was amplified from BS pig testis cDNA, yielding a sequence length of 1254 bp encoding 417 amino acids. Structural domains analysis unveiled PICK1 protein contained two conserved domains, the PDZ conserved domain, and the BAR-PICK1 conserved domain, playing pivotal roles in various cellular functions. The PDZ domain, highly conserved across species from mollusks to vertebrates, interacted with over 40 different ligands through their respective C-termini (Erlendsson and Madsen, 2015). Similarly, the BAR domain regulated the localization and function of target proteins (Peter et al., 2004), bound to lipid molecules, and associated with curved vesicle membranes, serving as a crucial domain for vesicle-to-acrosome transport. Moreover, the PICK1 protein formed homodimers through its BAR domain (Steinberg et al., 2006), with lipid binding positively regulated by its PDZ domain and negatively regulated by its C-terminal acidic domain (Jin et al., 2006). Moreover, *PICK1* exhibited high conservation across multiple animal species, with over 95% similarity observed compared to 17 other animals (Xu and Xia, 2006). Further, phylogenetic analysis indicated that BS pigs clustered with horses, indicating a high conservation in evolution.

Protein interaction analysis revealed that PICK1 interacted with 50 proteins. KEGG enrichment analysis indicated these proteins were involved in some vital pathways, including glutamatergic synapse, circadian entrainment, neuroactive ligand-receptor interaction, long-term depression, dopaminergic synapse, and synaptic vesicle cycle. Furthermore, GO annotation underscored their predominant involvement in molecular functions. Correlating these proteins with the gene expression from transcriptome data of BS, we found significant correlations between PICK1 and DLG4, as well as TBC1D20. DLG4, also known as PSD95, plays a pivotal role in brain development processes and influences susceptibility to post-premature birth injury. It is predominantly synthesized by microglial cells in immature mice and humans, regulated by developmental cues and inflammatory responses. Variations in DLG4 are associated with structural differences in the brains of preterm individuals (Krishnan et al., 2017). Functionally, PSD-95 is integral to organizing the postsynaptic density (PSD) structure, impacting synaptic maturation, dendrite morphology, and regulating NMDA and AMPA glutamate receptors. Perturbations in PSD-95 function due to missense variants can lead to DLG4-related synaptopathy (Sheng and Kim, 2011; Rodríguez-Palmero et al., 2021). TBC1D20, localized in the Golgi apparatus and endoplasmic reticulum, exhibited widespread expression across various reproductive cell subtypes and supporting cells. Its deficiency in mouse-supporting cells precipitates irreversible endoplasmic reticulum stress, prompting G1/S arrest and excessive apoptosis, ultimately resulting in infertility in 'blind sterile' male mice (Chang et al., 2019). TBC1D20 also maintains the integrity of the blood-testis barrier (BTB), crucial for supporting cell maturation and BTB integrity (Cui et al., 2020). Additionally, TBC1D20 regulates autophagosome maturation, facilitating the removal of damaged proteins and organelles in lens fiber cells to maintain lens transparency. In the testis, TBC1D20-mediated autophagosome maturation is essential for autophagic flux and acrosome formation. Human TBC1D20 dysfunction manifests Warburg Micro syndrome 4 (WARBM4), a rare autosomal recessive disorder characterized by congenital eye, brain, and genital abnormalities (Sidjanin et al., 2016).

MicroRNAs (miRNAs) are short, single-stranded non-coding RNAs typically comprising 19-22 nucleotides, originating from local hairpin structures processed by two RNase III enzymes, Drosha and Dicer. Functionally, miRNAs negatively regulate gene expression post-transcriptionally by binding to complementary sites within the 3’UTR region of target mRNAs (Kim et al., 2009). These molecules wield significant influence across diverse biological processes including development, apoptosis, proliferation, differentiation, transformation, and cellular senescence (Lujambio and Lowe, 2012; Gorospe and Abdelmohsen, 2011). Functional annotation of the porcine *PICK1* gene and construction of the ceRNA regulatory network revealed that *PICK1* was targeted by 7 miRNAs: ssc-miR-127, ssc-miR-330, ssc-miR-7134-5p, ssc-miR-744, ssc-miR-199a-5p, ssc-miR-370, and ssc-miR-423-5p. Among them, miR-127 stands out for its crucial role in cell proliferation, differentiation, and development. Notably, it targets BCL6 to regulate cell proliferation; and enhance myogenic cell differentiation by targeting S1PR3 (Chen et al., 2013; Zhai et al., 2017). In pig adipocytes, miR-127 regulates preadipocyte proliferation by inhibiting MAPK4 and suppresses preadipocyte differentiation into adipocytes by blocking HOXC6, thereby mitigating fat accumulation (Gao et al., 2019). Similarly, miR-330-5p negatively regulates sheep preadipocyte differentiation by targeting BCAT2, thus negatively regulating the differentiation of sheep preadipocytes (Shi et al., 2018). In addition, while miR-330-3p is implicated in various cancers such as non-small-cell lung cancer, laryngeal squamous cell carcinoma cells, gastric cancer, and ovarian cancer (Cai et al., 2021). Ssc-miR-7134-3p negatively regulates fat deposition in castrated boars by targeting MARK4 (Wang et al., 2017). MiR-744 enhances the type I interferon signaling pathway by targeting PTP1B in human renal glomerular cells and inhibits the proliferation, invasion, and migration by targeting SOX12/Wnt/β-catenin (Guo et al., 2021; Zhang et al., 2015). Moreover, miR-199a-5p promotes porcine preadipocytes and is associated with embryo implantation (Shi et al., 2014; Wang et al., 2013). ssc-miR-370 promotes porcine preadipocyte proliferation by facilitating the G1/S phase transition and inhibiting adipogenic differentiation by targeting FoxO1. (Chu et al., 2021). miR-423-5p inhibits myoblast proliferation and differentiation and regulates asthenozoospermic sperm, promoting oxidative stress and inhibiting sperm motility (Ge et al., 2018; Zhang et al., 2021).

Multi-tissue qPCR analysis revealed that *PICK1* exhibited extensive expression across various tissues in BS pigs, and was highly expressed in the urethral gland and testis. Notably, the PDZ-based interaction between PICK1 and GluR2, as well as GluR2 phosphorylation, are necessary for long-term depression expression in the cerebellum, a form of synaptic plasticity crucial for certain types of motor learning processes (Steinberg et al., 2006). *PICK1* in the pancreas is primarily involved in insulin production (Cao et al., 2013; Holst et al., 2013). Meanwhile, in the testis, it plays a significant part in acrosome formation during spermatogenesis, deficiency of which can lead to male infertility in mice (Xiao et al., 2009). Intriguingly, *PICK1* exhibited high expression not only in the testes but also in the urethral glands of BS pigs, prompting the need for further investigation into its function. At the cellular level, prior studies indicated that PICK1 was mainly located in the Golgi apparatus and secretory vesicles (Cao et al., 2013; Holst et al., 2013). However, our subcellular localization findings revealed predominantly cytoplasmic expression in ST cell lines, consistent with previous studies (Staudinger et al., 1995).

## Conclusion

The integration of long-read and short-read sequencing methodologies provided a comprehensive insight into the transcriptional regulation and expression of *PICK1*. Through an investigation into the transcriptional complexity arising from alternative splicing events within *PICK1*, transcript characteristics, ceRNA-mediated regulatory, and the expression profiles at both mRNA and cellular levels were elucidated. Notably, four distinct transcripts were identified, with ENSSSCT00000000120 exhibiting the most exons count (14 exons), and displaying a conserved amino acid sequence across 18 species. Additionally, 50 proteins interacting with PICK1 were delineated, mainly involving glutamate synapses and amphetamine synthesis. Addiction pathways, and neuroactive ligand-receptor interactions. Significant associations were observed between PICK1 and DLG4, TBC1D20. PICK1 was primarily involved in 9 GOs, encompassing seven cellular components and two molecular functions. Seven miRNAs were identified as regulators of *PICK1* expression. Substantial expression of *PICK1* was detected in the urethral glands and testes, predominantly localized within the cytoplasm of ST cells. These findings broaden our understanding of the transcriptional regulatory properties underlying the spermatogenesis-related gene *PICK1*, thereby laying the foundation for further elucidation of its function and molecular mechanism within the testes of BS pigs.
